# Determination of BRCAness Phenotype in Breast Tumors for the Appointment of Neoadjuvant Chemotherapy Based on Platinum and Taxanes

**DOI:** 10.3390/ijms24010207

**Published:** 2022-12-22

**Authors:** Matvey Mihajlovich Tsyganov, Marina K. Ibragimova, Evgeniy Y. Garbukov, Olga D. Bragina, Ariana A. Karchevskaya, Evgeny A. Usynin, Nikolai V. Litvyakov

**Affiliations:** 1 Department of Experimental Oncology, Research Institute of Oncology, Tomsk National Research Medical Center of the Russian Academy of Sciences, St. Kooperativny 5, Tomsk 634009, Russia; 2 Faculty of Medicine and Biology, Siberian State Medical University, 2 Moskovsky Trakt, Tomsk 634050, Russia; 3 Biological Institute, The National Research Tomsk State University, Prospekt Lenina, 36, Tomsk 634050, Russia

**Keywords:** BRCAness, homologous recombination, homologous recombination deficiency, breast cancer, gene expression, microarray analysis, DNA copy number aberration, loss of heterozygosity, neoadjuvant chemotherapy, personalized treatment

## Abstract

The concept of BRCAness was developed because of similarities between sporadic and hereditary breast cancer. BRCAness defines the pathogenesis and treatment sensitivity of many types of cancer, as well as the presence of a defect in the homologous recombination repair of tumor cells simulating the loss of *BRCA1* or *BRCA2*, as in the presence of germline mutations. The question of treatment effectiveness for BRCA-like tumors is controversial and open. Thus, the aim of this work was to study the effectiveness of neoadjuvant chemotherapy (NAC) in BRCA-deficient breast cancer patients without germline mutations. The study involved 130 patients with breast cancer in stages IIA–IIIB. The treatment regimen included neoadjuvant chemotherapy, surgery, and adjuvant chemotherapy. The materials used were tumor samples from before and after chemotherapy. DNA and RNA were isolated from the tumor material. RNA was used to assess the expression level of *BRCA1*, while DNA was used for methyl-sensitive PCR. A microarray analysis was performed on high-density DNA chips from an Affymetrix CytoScan^TM^ HD Array to assess DNA copy number aberration (CNA status) and loss of heterozygosity. A statistical analysis was performed using the Statistica 8.0 application package. It was noted that the existence of copy number aberrations in genes was statistically significantly associated with tumor treatment response and disease prognosis. Patients with partial regression had a statistically significantly higher amount of deletion than patients without an objective response (5/25 patients; 16%), as shown in the general sample of patients (52.9% versus 27.1%, respectively) at *p* = 0.0001 and in patients treated with anthracycline-containing regimen (*p* = 0.0001). In addition, it was shown that patients with *BRCA1* deletion had higher rates of metastatic-free survival (log rank test, *p* = 0.009). BRCAness patients had a higher rate of 5-year metastatic survival, but not of treatment efficacy. The prospective study showed the positive effect of assessing the BRCAness phenotype of a tumor before treatment and of prescribing personalized NAC regimens. The objective response rate was statistically significantly more often observed in the group of patients with personalized chemotherapy (85.0% (34/40 patients) versus 62.3% (56/90 patients); *p* = 0.007). Despite the controversial effectiveness of BRCA-like tumor treatment, our data showed high predictive and prognostic significance of the BRCAness phenotype for the personalization of platinum and taxane regimens.

## 1. Introduction

Patients with heterozygous germline mutations of the *BRCA1* or *BRCA2* genes (for example, c.5266dupC, c.1961del, c.3700_3704del, c.3756_3759del, c.4035del in *BRCA1*, c.5946del in *BRCA2*, etc.) have an increased risk of developing breast cancer (BC), as well as ovarian and other cancers [1]. The *BRCA1* gene plays an important role in cells, as it is associated with a number of cellular processes, such as DNA repair, the regulation of transcription, and chromatin remodeling. *BRCA2* function is limited by the DNA repair and recombination processes and is important in the regulation of *RAD51* activity [2].

Cells with *BRCA1* or *BRCA2* dysfunction have a deficiency in double-stranded DNA breakage repair by the conservative mechanism of homologous recombination (HR). Homologous recombination deficiency (HRD) allows DNA damage by nonconservative, potentially mutagenic mechanisms, such as nonhomologous end-joining and single-strand annealing, which can lead to genomic instability and the risk of malignancy. This phenomenon is reflected in the effectiveness of treatment and the sensitivity of the drugs used [3].

In two clinical trials conducted in patients with breast cancer treated with neoadjuvant monotherapy using cisplatin, the incidences of complete regression in patients with *BRCA1* germline mutations were 100% and 83%, respectively [4]. Tumor cell lines lacking functional *BRCA1* and *BRCA2* genes had increased sensitivity to platinum and DNA-damaging agents, such as anthracyclines [5].

The process of HRD in sporadic forms of cancer can be caused not only by the presence of germline mutations, but also by other mechanisms. *BRCA1*-similar tumors have similar properties and are defined as “BRCAness” (BRCA-like tumors), and these may be important for treatment [6]. The concept of BRCAness defines the pathogenesis and treatment sensitivity of many types of cancer. BRCAness is the presence of a defect in the homologous recombination repair of tumor cells mimicking the loss of *BRCA1* or *BRCA2*, as well as germline mutations [7]. The BRCAness concept was developed due to similarities between sporadic breast cancer and familial *BRCA1* cancer [8]. In sporadic cancer, *BRCA1* is inactivated by other mechanisms, such as the aberrant methylation of cytosine residues in CpG dinucleotides, low expressions of protein and mRNA, loss of heterozygosity, etc. The BRCAness phenotype is associated with the most aggressive clinical and pathological features of tumors: large tumor size (>2 cm, at *p* = 0.009), the presence of lymphogenous metastasis (*p* = 0.008), stage 3 of the disease (*p* = 0.001), and high levels of Ki-67 (*p* = 0.001) [9]. The issue of the relationships of the BRCAness phenotype with the effectiveness of treatment and prognosis is still controversial. Many studies have indicated that epithelial ovarian cancer patients with zero or low *BRCA1* protein expressions have a better chance of clinical response after chemotherapy compared with patients with high *BRCA1* expressions (HR: 2.47; 95% CI: 1.10–5.55; *p* = 0.029). Patients with low expressions of *BRCA1* showed clinical responses after monotherapy with platinum-containing drugs compared to patients with high expressions (68.5% vs. 46.8%) [10]. Another study showed that there were no significant differences in pCR or relapse rates between BRCAness and non-BRCAness groups. Patients in the non-BRCAness group tended to achieve pCR more often than patients in the BRCAness group (58.1% versus 39.2%, respectively; *p* < 0.1). In addition, the BRCAness group had a lower disease-free survival rate [11]. A comparative study on the efficacies of carboplatin and docetaxel in patients with familial breast cancer and BRCA-like tumors was carried out. BRCA-associated patients achieved statistically significantly higher efficacy with carboplatin than with docetaxel (68% versus 33%, respectively; *p* = 0.01). At the same time, a similar effect was not obtained for BRCAness tumors with methylated gene promoters and low or absent expressions [12]. It is important to note that the presence of *BRCA1* or *BRCA2* deficiency is also associated with the effectiveness of other chemotherapy drugs. In particular, in vitro experiments showed strong selective activities of chlorambucil, melphalan, and nimustine against *BRCA2*-deficient cells and specific synergism with olaparib [13]. A recent study also showed that melphalan, as a bifunctional alkylating agent, was selectively effective in BRCA-deficient epithelial ovarian cancer. It was found that *BRCA1-* and *BRCA2*-mutant patients showed significantly longer progression-free survival (PFS) rates compared to *BRCA1* and -*2* wild-type patients (6.2 versus 2.6 months, respectively; hazard ratio (HR): 0.25; 95% confidence interval (CI): 0.10–0.61; *p* = 0.002). Moreover, a trend of better overall survival (OS) was seen for *BRCA1-* and *BRCA2*-mutant patients (25.9 versus 8.0 months; HR: 0.38; 95% CI: 0.12–1.19; *p* = 0.097) [14].

The aim of this work is to study the effectiveness of neoadjuvant chemotherapy and disease prognosis in BRCA-deficient breast cancer patients without germline mutations.

## 2. Results

### 2.1. Retrospective Study

A total of 130 patients were analyzed in this study (Table 1), of which 90 patients made up the retrospective group and 40 patients made up the prospective group. There were no significant differences between the original characteristics of the groups of patients treated with either a personalized approach or standard chemotherapy (Table 1). Statistically significant differences were found only for the menstrual statuses of the patients, with premenopause observed mainly in the group with personalized chemotherapy (82.5%, 33/40 cases, Fisher’s exact probability test, *p* = 0.005). In addition, the group treated with personalized NAC treatment consisted of patients with luminal-B-subtype BC (92.5%, 37/40 patients). It should be noted that a comparison of the tumor response rates to neoadjuvant chemotherapy showed that an objective response (complete or partial tumor regression) was statistically significantly more frequent in the second group of patients (85.0% (34/40 patients) versus 62.3% (56/90 patients); *p* = 0.007). Details are presented in Table 1.

As a result of the study, we assessed the expression of the *BRCA1* gene in a retrospective group of patients before NAC and the methylation status of the promoter region of this gene, as well as the use of a microarray study, the frequency of copy number aberrations in this gene, and the presence of areas of loss of heterozygosity.

**Figure 1:**
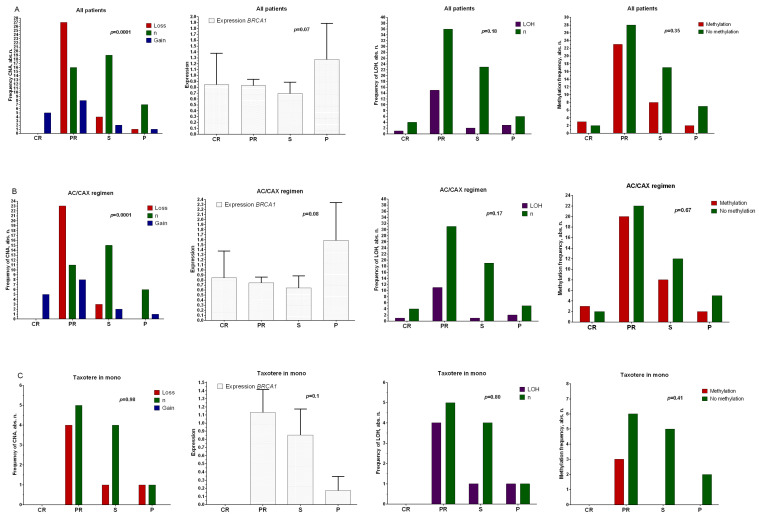
Relationships between CNA, gene expression, sites of loss of heterozygosity, and methylation of the promoter region of the *BRCA1* gene with the effectiveness of NAC in a general group of patients (**A**), those treated with anthracycline-containing regimen (AC/CAX) (**B**), and those treated with taxotere monotherapy (**C**). Note: abscissas show the effect of neoadjuvant chemotherapy. CR: complete regression; PR: partial regression; ST: stabilization; P: progression. A nonparametric Kruskal–Wallis test was used to assess the differences among independent samples in terms of expression level. Frequency comparisons are based on two-tailed Fisher’s test or chi-squared test.

The analysis showed that no statistically significant differences were found between the studied parameters of the *BRCA1* gene and the main clinical and morphological parameters. It should be noted that the frequency of *BRCA1* gene copy number aberrations in breast tumors was 53.3% (48/90 cases). The frequency of deletions was 35.6% (32/90 cases); the highest frequency of deletions (54.5%) was observed in HER2+ breast cancer. The literature data indicates that hereditary *BRCA1* gene mutation is associated with damage in the DNA repair system and the high efficiency of DNA-damaging agents, such as platinum-based preparations. It can be assumed that the deletion of the *BRCA1* gene locus, as well as its low expression, can cause HRD and is associated with high sensitivity of the neoplasm to platinum preparations and DNA-damaging agents. We analyzed the relationships between the effect of neoadjuvant chemotherapy and the expression level and DNA copy number aberration (CNA status) of the *BRCA1* gene in retrospective patients (Figure 1).

Figure 1A shows that the deletion frequency in patients with partial regression was statistically significantly higher (27/51 patients, 52.9%, Fisher’s exact probability test, *p* = 0.0001) than those of the groups of patients with stabilization (frequency of deletions: 16%, (4/25 patients)) and progression (frequency of deletions: 11.1% (1/9 of patients)) in the general group of BC patients. A similar result was found for patients treated with the anthracycline-containing NAC (AC/CAX) regimen (Figure 1B), with over 50% of patients with *BRCA1* deletion displaying an objective response to the treatment (Fisher’s exact probability test, *p* = 0.0001). No correlation was shown between the studied parameters and the effectiveness of NAC in patients treated with taxotere (Figure 1C). The association of chemotherapy efficacy with expression did not show statistically significant differences. An association was shown at the tendency level: in the general group of patients (Figure 1A) and in those treated with the anthracycline-containing regimen (Figure 1B), the levels of *BRCA1* expression were higher in patients with tumor progression during treatment compared with the group of patients showing objective treatment responses (*p* = 0.07). There was no relationship between either the presence of sites of loss of heterozygosity or methylation of the promoter region of the *BRCA1* gene and the effectiveness of NAC in any of the studied groups.

**Figure 2:**
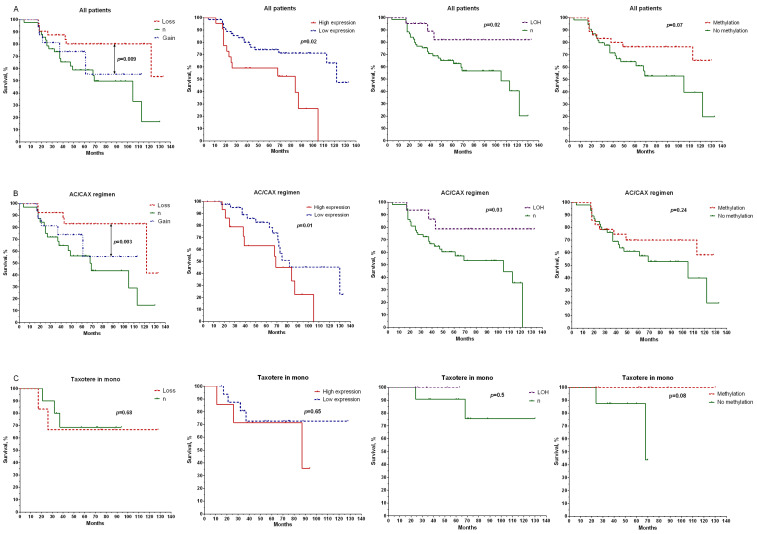
Relationships between CNA, gene expression, sites of loss of heterozygosity, and methylation of the promoter region of the BRCA1 gene with metastatic-free survival in the general group of patients (**A**), patients treated with anthracycline-containing regimen (AC/CAX) (**B**), and patients treated with taxotere monotherapy (**C**). Note: a log rank test was used to compare significance of differences among groups in terms of survival.

An analysis of the metastatic-free survival of patients with copy number aberrations in the *BRCA1* gene showed that the presence of deletion caused a higher survival rate (log rank test, *p* = 0.009) than those of the group of patients with normal gene states (Figure 2A), for the entire group of patients, and for the AC/CAX group, with a log rank test result of *p* = 0.003 (Figure 2B).

In addition, it was shown that the metastatic-free survival (MFS) values were higher in the group of patients with low expressions of the studied gene (log rank test, *p* = 0.02), as well as in patients who had losses in *BRCA1* heterozygosity (log rank test, *p* = 0.02) (Figure 2A). In the first case, the rate of 10-year MFS was 64% compared with the group of patients with gene overexpressions. The 10-year survival rate was 82% for patients with LOH versus 40% in the group with normal gene states. The relationship between survival rate and methylation status of the *BRCA1* gene promoter did not show a statistically significant difference. Only a relationship with the general group of patients at the level of a pronounced trend was shown (Figure 2A).

We evaluated the effect of the BRCAness phenotype on treatment efficacy and disease prognosis. If patients had deletion, hypoexpression of the *BRCA1* gene (expression less than 1), or a loss of heterozygosity, they were grouped as deficient in homologous recombination (BRCAness), while other patients were grouped as normal *BRCA1* gene functional activity (non-BRCAness). The BRCAness group included 75% of the patients (68/90 cases).

**Figure 3:**
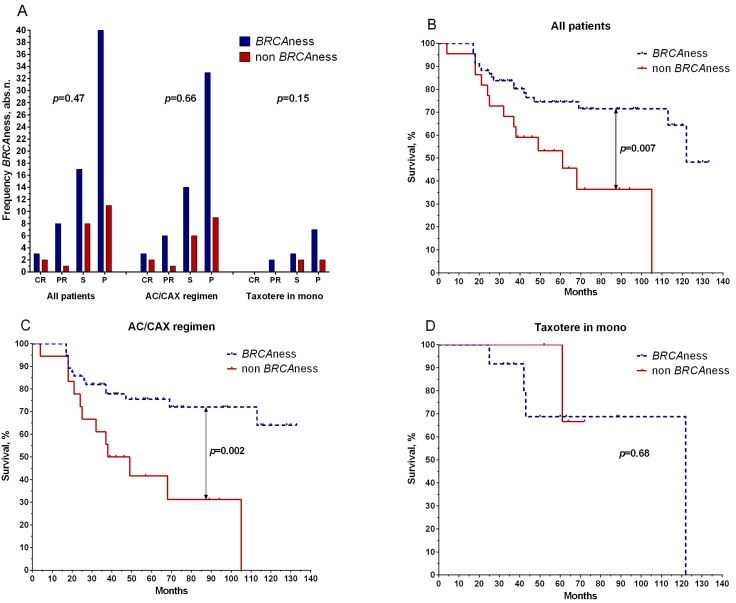
Relationship between BRCAness phenotype with effectiveness of NAC (**A**) and metastatic survival in the general group of patients (**B**), patients treated with anthracycline-containing regimen (AC/CAX) (**C**), and patients treated with taxotere monotherapy (**D**). CR: complete regression; PR: partial regression; ST: stabilization; P: progression. Wilcoxon–Mann–Whitney test was used to test significance of differences among the study groups. Frequency comparisons were based on two-tailed Fisher’s test or chi-squared test.

Figure 3 shows that the frequencies of patients with the BRCAness and non-BRCAness phenotypes did not differ in effectiveness and regimens of neoadjuvant chemotherapy (Figure 3A).

Patients with BRCAness phenotypes had higher 5-year metastatic-free survival rates (75%) in the general group (log rank test, *p* = 0.007) than non-BRCAness patients (53%) (Figure 2B). Patients with the AC/CAX treatment scheme showed a similar result: the 5-year metastatic-free survival of patients with BRCAness phenotypes was 76% versus 42% for those without, with a log rank test result of *p* = 0.002 (Figure 2C). No statistically significant differences in the survival rate were found in patients treated with the taxotere monoregimen (log rank test, *p* = 0.68) (Figure 3D).

### 2.2. Prospective Study

**Figure 4:**
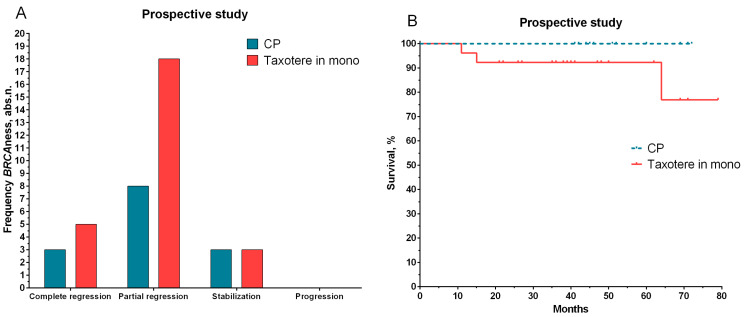
The effect of NAC (**A**) and metastatic survival rates (**B**) for patients with personalized treatment depending on status of the *BRCA1* gene. Note: log rank test was used to compare the significance of differences among groups in terms of survival.

We conducted a prospective study on the significance of *BRCA1* status for personalized NAC regimens based on our data and the literature. The prospective group included 40 patients (Table 1). If the patients had low expressions or deletions of the *BRCA1* gene (presence of a deficiency in homologous recombination), they received NAC regimens with platinum-based preparations (cyclophosphamide at 600 mg/m^2^ on the first day and cisplatin at 100 mg/m^2^ on the first day (CP)). The rest of the patient group received taxotere (100 mg/m^2^ hourly infusion per day). For the group of patients treated with CP, 78.6% had an objective response to the treatment, including complete and partial tumor regression (11/14 patients). A similar result was shown for patients in the second group (taxotere) for complete and partial regression (88.5%, 23 out of 26 patients) (Figure 4).

The median follow-up among the patients included in the study was 32.0 months. The median follow-up among patients in the first group was 49 months (range of 41–72 months), and the follow-up is ongoing. Thus far, all patients have displayed 100% metastatic-free survival (Figure 4B). In three patients treated with taxotere (median of 41 months in the range of 11–79 months), the development of metastatic disease was observed at 11, 15, and 64 months.

## 3. Discussion

The present study found that *BRCA1* gene expression weakly affected the results of neoadjuvant chemotherapy in breast cancer patients, but the presence of copy number aberrations was statistically significantly associated with the tumor response to treatment. Thus, the presence of deletions in patients with partial regression was statistically significantly higher than in patients with stabilization and progression, both in the general sample of patients and in patients treated with the anthracycline-containing regimen. In addition, patients with a deletion of the 17q21.31 locus had a higher rate of metastatic-free survival.

Evaluation of the effect of the BRCAness phenotype on the effectiveness of treatment and disease prognosis made it possible to establish that patients with BRCAness phenotypes had a higher rate of 5-year metastatic-free survival. At the same time, the prospective study proved the positive effect of the BRCAness phenotype on tumors before treatment, as well as for patients prescribed personalized NAC treatments.

Clinical studies on the effect of BRCAness on treatment effectiveness and tactics showed that HRD status and its assessment predicted pCR in patients with triple-negative breast cancer (*p* = 0.0012) [15]. In addition, for GeparSixto (GBG 66), it was found that the pCR rate in patients with triple-negative breast cancer in a general group (regardless of *BRCA1* or -*2* status) was higher than that in a carboplatin-treated group (RR: 1.87; 95% CI: 1.17–2, 97; *p* = 0.009), with 56.8% (83 of 146 patients) in the carboplatin group and 41.4% (60 of 145) in the carboplatin-free group. Moreover, tumors with HRD were more likely to reach pCR than tumors with high HR (55.9% versus 29.8%, *p* = 0.001). Patients with HRD tumors showed a higher pCR rate when carboplatin was added to the chemotherapy regimen (64.9% vs. 45.2%; *p* = 0.025) [16]. Subsequently, Quereda V. et al. showed that SR-4835, a recently developed specific inhibitor of CDK12/13, sensitized triple-negative breast cancer cells to PARP inhibitors and DNA-damaging chemotherapeutic drugs by reducing the expressions of homologous recombination genes, specifically *BRCA1* and -*2* [17]. PARP inhibitors (PARPis) are the first clinically approved drugs designed to exploit synthetic lethality in tumors harboring *BRCA1* and -*2* mutations. Recent evidence indicated that PARPis had the potential to be used both in monotherapy and combination strategies of breast cancer treatment [18]. Thus, in 2010, a study was conducted of patients with locally advanced breast cancer with and without *BRCA1* and -*2* mutations. After treatment with olaporib, 41% (11 of 27 patients) of patients in the first group with the presence of mutations responded to therapy: one patient had a complete pathological response and ten had partial responses, while 22% (6 of 27 patients) responded to the therapy. In the second group (without disorders in the *BRCA1* and *BRCA2* genes), a complete response was not observed [19]. A statistically significant increase in PFS from 5.6 to 8.6 months was also shown, and the objective response rate was twice as high in patients treated with talazoparib (a poly-ADP ribose polymerase (PARP) inhibitor), at 62.6% and 27.2%, respectively, for groups of patients with *BRCA1* and -*2* mutations and without mutations [20]. Similar results have been shown for other nonspecific localizations. Therefore, preclinical studies have suggested that certain cancers, such as biliary tract cancer [21], pancreatic cancer [22], etc., are susceptible to PARP inhibition in the presence of homologous recombination deficiency.

Regarding the use of taxanes in patients with BRCAness, a 2022 study by Liu L. et al. showed that the clinical response rate to taxane chemotherapy was significantly lower in patients with BRCAness (58.3% vs. 77.8%; *p* = 0.041) compared with a group of patients without this phenomenon. In addition, the authors also showed that such patients had higher rates of 5-year disease-free (54.0% vs. 88.0%; *p* < 0.001) and overall survival (76.3% vs. 93.1%; *p* = 0.002) [23]. A similar study was conducted for anthracycline-containing HCT regimens, but an interim analysis showed that the rate of complete pathological response was lower than calculated, which could be due to the nonspecificity of BRCAness to anthracycline-containing regimens [24].

*BRCA1* expression is correlated with the hypermethylation status of the gene promoter, and both are associated with the prognosis and efficacy of chemotherapy. The five-year survival rates were 79.9% and 71% (*p* = 0.021) for groups of patients with hypermethylated and nonhypermethylated *BRCA1* promoters, respectively [25]. However, a study showed that BRCAness was not a matter of major significance in predicting the effect of platinum-based breast cancer treatments on PARP inhibitors [26]. Some studies indicate the opposite effect: patients with BRCAness profiles had higher rates of relapse-free (34 vs. 15 months; *p* = 0.013) and overall survival (72 vs. 41 months; *p* = 0.006) compared with patients with profiles other than BRCA-like cancer [27].

Thus, while the identification of *BRCA1* and -*2* germline mutations associated with susceptibility to platinum drugs or PARP inhibitors can help select patients, another challenge is the identification of patients who may be sensitive to these drug classes despite the presence of wild-type *BRCA1* and -*2* genes at the constitutional level. In this case, the assessment of BRCAness phenotype, in which molecular changes in a tumor lead to disruption of the HR repair process, can further expand the patient population potentially suitable for DNA-damaging chemotherapy. However, this putative patient population and its molecular features have yet to be clearly identified. First, the expressions of *BRCA1* and -*2* genes in a tumor can be suppressed by hypermethylation of the gene promoter [28], or somatic mutations and large chromosomal rearrangements can be found in these genes [29], although the impact of these changes on PARP inhibitors and platinum sensitivity remains unclear. In addition, a number of individual genes that are also known to be involved in HR repair, such as *ATM*, *ATR*, *CHEK1*, *CHEK2*, *RAD51*, *NBS1*, *FANCE*, *CDK12*, *ERCC1*, *PTEN*, *BRIP1*, etc., may show abnormalities (germ or somatic) that are also associated with susceptibility to DNA-damaging agents [6].

Thus, the results obtained in the course of this research allowed us to formulate a number of definitions and new provisions for the possibility of using *BRCA1* gene parameters for a personalized approach to the treatment of breast cancer.

The predictive and prognostic significance of *BRCA1* gene parameters were assessed. It was established that the presence of a deletion and low expression had high predictive values for treatment. It was shown that the presence of these *BRCA1*-aberrant states, as well as the presence of germline mutations, caused the formation of a deficiency in homologous recombination and could be used as a marker for a personalized approach to prescribing chemotherapy in patients without germline mutations. Based on the results of the prospective study, it can be stated that a preliminary assessment of *BRCA1* expression and the presence of a deletion in the biopsy material of a breast tumor was a good predictive and prognostic marker for prescribing treatment. The significance of their clinical application from the point of view of the advisability of prescribing preoperative chemotherapy to patients with breast cancer according to schemes with the inclusion of platinum and taxane preparations was shown.

## 4. Materials and Methods

The study involved 130 breast cancer patients at stages IIA–IIIB with morphologically verified diagnoses aged 27–68, with the average age being 48.1 ± 0.9 years (mean ± SE) (Table 1). The retrospective study involved 90 patients. In accordance with the Consensus Conference on Neoadjuvant Chemotherapy in Carcinoma of the Breast, April 26–28, 2003, Philadelphia, Pennsylvania [30], all patients received 2–8 courses of neoadjuvant chemotherapy: AC (doxorubicin at 60 mg/m^2^ intravenously on the first day and cyclophosphamide at 600 mg/m^2^ intravenously on the first day) and CAX (cyclophosphamide at 100 mg/m^2^ intravenously on the 1st–14th days, doxorubicin at 30 mg/m^2^ intravenously on the 1st and 8th days, and xeloda at 2000 mg/m^2^ orally on the 1st–14th days) or taxotere monotherapy (100 mg/m^2^ hourly infusion per day). Operations were performed 3–5 weeks after NAC treatment. Then, the patients received 2 courses of adjuvant chemotherapy according to the FAC regimens, and radiation therapy and hormonal treatment were prescribed as indicated. The prospective study involved 40 patients with personalized NAC regimens: CP (cyclophosphamide) at 600 mg/m^2^ on the 1st day and cisplatin at 100 mg/m^2^ on the 1st day) or taxotere monotherapy (100 mg/m^2^ hourly infusion per day). The study was conducted according to the ethical principles suggested in the Declaration of Helsinki (fixed in 2013) and approved by the Ethical Committee of the Tomsk Cancer Research Institute (14 January 2013 protocol). All patients did not have frequent germline mutations in the *BRCA1* and *BRCA2* genes. We analyzed biopsy tumor samples before treatment (~10 mm^3^ volume), which were obtained under the control of an ultrasound, as well as surgical samples after NAC treatment (~30–90 mm^3^ volume). Tumor samples were placed in RNAlater solution (Thermo Scientific, Waltham, MA, USA) and stored at −80 °C (after 24 h of incubation at +4 °C) for further DNA isolation.

### 4.1. RNA Isolation

RNA was isolated from 130 tumor samples before treatment using an RNeasy mini kit Plus containing DNase I (Qiagen, Germany) and Ribolock RNAse inhibitor (Thermo scientific, Waltham, MA, USA). The concentration and purity of RNA isolation were assessed with a NanoDrop-2000 spectrophotometer (Thermo Scientific, Waltham, MA, USA; 56–120 ng/μL; A_260_/A_280_ = 1.75–1.85; A_260_/A_230_ = 1.75–2.00). The RIN (RNA integrity number) was 6.4–7.9. The algorithm for assigning RNA integrity values was detailed by Schroeder A. et al. in 2006 [31]. To obtain cDNA on an RNA template, a reverse transcription reaction was performed using a RevertAid ™ kit (Thermo scientific, Waltham, MA, USA) with random hexanucleotides.

### 4.2. Quantitative PCR

The *BRCA1* gene level expression (PubMed NM_007294.3) was assessed using reverse-transcriptase quantitative real-time PCR (RT-qPCR) with original primers and probes using TaqMan technology (*forward primer*: 5′-acagctgtgtggtgcttctgtg-3′; *reverse primer*: 5′-cattgtcctctgtccaggcatc-3′; *probe*: FAM-5′-catcattcacccttggcacaggtgt-3′-BHQ1; amplicon: 107 bp) with a Rotor-Gene-6000 instrument (Corbett Research, Australia). PCR was performed three times in a 15 μL total volume containing 250 μM dNTPs (Sibenzyme, Russia), 300 nM forward and reverse primers, 200 nM probe, 2.5 mM MgCl_2_, 19 SE buffer (67 mM Tris-HCl (pH 8.8 at 25 °C), 16.6 mM (NH4) 2SO4, and 0.01% Tween-20), 2.5 units HotStart Taq polymerase (Sibenzyme, Russia), and 50 ng cDNA. The two-step amplification program included 1 cycle at 94 °C for 10 min of preliminary denaturation and 40 cycles of step 1 at 94 °C for 10 s and step 2 for 20 sec at a temperature of 60 °C. *GAPDH* (glyceraldehydes-3-phosphatedehydrogenase) and *ACTB* (actin beta) were used as reference genes. The level of gene expression was normalized to the expressions of the reference genes and was measured in arbitrary units [32]. Pooled RNA from 20 patients was isolated from the normal breast tissue of patients who did not undergo NAC.

### 4.3. DNA Extraction

DNA was isolated from 130 samples of tumor tissue using a QIAamp DNA mini Kit (Qiagen, Venlo, Netherlands). DNA concentration and purity of isolation were evaluated with a NanoDrop-2000 spectrophotometer (Thermo Scientific, Waltham, MA, USA) (from 50 to 190 ng/μL; A_260_/A_280_ = 2.05–2.20; A_260_/A_230_ = 1.95–2.20). DNA integrity was assessed by capillary electrophoresis with a TapeStation instrument (Agilent Technologies, Santa Clara, CA, USA); DNA fragments had a mass of more than 60 kbp.

### 4.4. Methyl-Sensitive PCR

This method was based on the ability of methyl-sensitive restrictases to cleave (hydrolyze) CpG sections of DNA that did not undergo methylation and to leave uncleaved 5mCG sections containing methylcytosine. HpaII (C↓CGG) and HspaI (CGC↓G) (Sybenzym, Russia) were used as methyl-sensitive restriction enzymes. All restriction enzymes were used at a 5-fold excess (5 units of restriction enzymes were added to 1 µg of DNA). DNA with restriction enzymes was incubated for 14 h, and another 2.5 units of restriction enzyme activity were added, as well as HpaII (C↓CGG) with SE-B buffer (10 mM Tris-HCl (pH 7.6 at 25 °C), 10 mM MgCl2, and 1 mM DTT) and HspaI (CGC↓G) with SE-Y buffer (33 mM Tris-acetate (pH 7.9 at 25 °C), 10 mM magnesium acetate, 66 mM potassium acetate, and 1 mM DTT). This was followed by PCR with enzyme-treated DNA restriction as a matrix, with primers to the promoter of the target gene. These primers contained a restriction endonuclease recognition site, so the fragment under study was amplified only in the absence of DNA cleavage at this site. Primers were selected using the Vector NTI 11.5 program (Invitrogen, USA). The nucleotide sequences of the promoter regions of the studied genes were found in the Transcriptional Regulatory Element Database.

### 4.5. Microarray Analysis

A microarray analysis was performed on high-density microarrays (DNA chips) of an Affymetrix (USA) CytoScan^TM^ HD Array to determine the presence of chromosomal aberrations of the *BRCA1* gene. Sample preparation, hybridization, and scanning procedures were performed according to the manufacturer’s protocol of an Affymetrix GeneChip^®^ Scanner 3000 7G system (Affymetrix, Santa Clara, CA, USA). To process the results of microchipping, we used Chromosome Analysis Suite 4.0 (Affymetrix, Santa Clara, CA, USA) for the detection of aberrations—deletions or amplifications (losses or gains)—and loss of heterozygosity (LOH).

### 4.6. Statistical Methods

Statistical data processing was conducted using the Statistica 8.0 application package (StatSoft Inc., Tulsa, OK, USA). A Wilcoxon–Mann–Whitney test was used to test the significance of differences among the study groups. Differences were statistically significant at a significance level of *p* < 0.05. Metastatic-free survival (MFS) curves were constructed using the Kaplan–Meier method. A log-rank test was used to compare the significance of differences among the groups in terms of survival. Frequency comparisons were based on a two-tailed Fisher’s test or a chi-squared test.

## Figures and Tables

**Table 1:** Clinical and pathological characteristics of breast cancer patients.

Clinical and Pathological Parameter		Retrospective Group (n = 90); Number of Patients (abs. p. (%))	Prospective Group (n = 40); Number of Patients (abs. p. (%))	*p*-Value
Age	≤45	35 (38.9)	22 (55.0)	0.12
	>45	55 (61.1)	18 (45.0)	
Menstrual status	Premenopause	51 (56.7)	33 (82.5)	0.005 *
	Postmenopause	39 (43.3)	7 (17.5)	
Tumor size	T_1_	12 (13.3)	5 (12.5)	0.38
	T_2_	66 (73.3)	30 (75.0)	
	T_3_	4 (4.4)	4 (10.0)	
	T_4_	8 (8.9)	1 (2.5)	
Lymphogenous metastasis	N_0_	38 (42.2)	16 (40.0)	0.46
	N_1_	37 (41.1)	20 (50.0)	
	N_2_	6 (6.7)	3 (7.5)	
	N_3_	9 (10.0)	1 (2.5)	
Molecular subtype	Luminal B	60 (66.7)	37 (92.5)	0.007 *
	Triple-negative	19 (21.1)	2 (5.0)	
	HER2-positive	11 (12.2)	1 (2.5)	
Histological form	Unicentric	58 (64.4)	21 (52.5)	0.24
	Multicentric	32 (35.6)	19 (47.5)	
NAC regimen	CAX	28 (31.1)	-	-
	AC	46 (51.1)	-	
	Taxotere	16 (17.8)	26 (65.0)	
	CP	-	14 (35.0)	
NAC response	Progression	9 (10.0)	0 (0.0)	0.007 *
	Stabilization	25 (27.8)	6 (15.0)	
	Partial regression	51 (56.7)	26 (65.0)	
	Complete regression	5 (5.6)	8 (20.0)	

Note: * indicates significance for two-tailed Fisher’s probability test; CAX: cyclophosphamide, doxorubicin, and xeloda treatment; AC: doxorubicin and cyclophosphamide treatment; CP: cyclophosphamide and cisplatin treatment.

## Data Availability

Certificate of state registration of the database No. 2022621615 dated July 6, 2022 “Database of the results of sequencing of the *BRCA1* and *BRCA2* genes in patients with luminal B breast cancer treated with platinum preparations” Tsyganov M.M., Ibragimova M.K., Garbukov E.Yu., Usynin E.A., Litvyakov N.V. Certificate of state registration of the database No. 2022620759 dated April 6, 2022 “Database of the genetic landscape of breast cancer patients with a triple negative phenotype” Ibragimova M.K., Tsyganov M.M., Garbukov E.Yu., Zdereva E.A., Usynin E.A., Litvyakov N.V.
